# Multiplex Real-Time Reverse Transcription PCR for Influenza A Virus, Influenza B Virus, and Severe Acute Respiratory Syndrome Coronavirus 2

**DOI:** 10.3201/eid2707.210462

**Published:** 2021-07

**Authors:** Bo Shu, Marie K. Kirby, William G. Davis, Christine Warnes, Jimma Liddell, Ji Liu, Kai-Hui Wu, Norman Hassell, Alvaro J. Benitez, Malania M. Wilson, Matthew W. Keller, Benjamin L. Rambo-Martin, Yamundow Camara, Jörn Winter, Rebecca J. Kondor, Bin Zhou, Stacey Spies, Laura E. Rose, Jonas M. Winchell, Brandi M. Limbago, David E. Wentworth, John R. Barnes

**Affiliations:** Centers for Disease Control and Prevention, Atlanta, Georgia, USA (B. Shu, M.K. Kirby, W.G. Davis, C. Warnes, J. Liddell, J. Liu, K.-H. Wu, N. Hassell, A.J. Benitez, M.M. Wilson, M.W. Keller, B.L. Rambo-Martin, Y. Camara, J. Winter, R.J. Kondor, B. Zhou, S. Spies, L.E. Rose, J.M. Winchell, B.M. Limbago, D.E. Wentworth, J.R. Barnes);; Battelle Memorial Institute, Atlanta (W.G. Davis, J. Liddell); Leidos Inc, Atlanta (Y. Camara)

**Keywords:** SARS-CoV-2, influenza, COVID-19, multiplex, Flu SC2 Multiplex, acute respiratory infections, respiratory infections, severe acute respiratory syndrome coronavirus 2, 2019 novel coronavirus disease, coronavirus disease, zoonoses, viruses, coronaviruses, real-time RT-PCR, influenza A, influenza B

## Abstract

Severe acute respiratory syndrome coronavirus 2 (SARS-CoV-2) emerged in late 2019, and the outbreak rapidly evolved into the current coronavirus disease pandemic. SARS-CoV-2 is a respiratory virus that causes symptoms similar to those caused by influenza A and B viruses. On July 2, 2020, the US Food and Drug Administration granted emergency use authorization for in vitro diagnostic use of the Influenza SARS-CoV-2 Multiplex Assay. This assay detects influenza A virus at 10^2.0^, influenza B virus at 10^2.2^, and SARS-CoV-2 at 10^0.3^ 50% tissue culture or egg infectious dose, or as few as 5 RNA copies/reaction. The simultaneous detection and differentiation of these 3 major pathogens increases overall testing capacity, conserves resources, identifies co-infections, and enables efficient surveillance of influenza viruses and SARS-CoV-2.

An outbreak of pneumonia of unknown etiology in Wuhan, China, was reported to the World Health Organization on December 31, 2019 (*1*). Researchers determined that the illness, later known as coronavirus disease (COVID-19), was caused by a previously unidentified betacoronavirus, severe acute respiratory syndrome coronavirus 2 (SARS-CoV-2) (*2*). SARS-CoV-2 rapidly spread around the world, and on March 11, 2020, the World Health Organization declared a pandemic (*3*). By January 2021, SARS-CoV-2 had infected >96 million persons and caused >2 million deaths worldwide (*4*).

The high demand for molecular testing for SARS-CoV-2 has contributed to global shortages of diagnostic resources, including reagents, enzymes used in reverse transcription PCR (RT-PCR), plastic consumables, and staff availability (*5*,*6*). Efficient diagnostic tests can reduce strain on the testing system and decrease turnaround time. To improve testing efficiency, we developed the Centers for Disease Control and Prevention (CDC) Influenza SARS-CoV-2 (Flu SC2) Multiplex Assay, which is selective for influenza A and B viruses and SARS-CoV-2. This quadruplex real-time RT-PCR (rRT-PCR) simultaneously detects and distinguishes RNA of influenza A virus, influenza B virus, and SARS-CoV-2 in upper and lower respiratory specimens. To monitor specimen quality control, the assay also detects the *Homo sapiens* (human) RNase P (RP) gene. Because the Flu SC2 Multiplex Assay can test 93 samples in a 96-well plate, this technology improves the throughput of SARS-CoV-2 testing by 3-fold compared with the CDC 2019-nCoV Real-Time RT-PCR Diagnostic Panel (*7*). The Flu SC2 Multiplex Assay also simultaneously detects influenza A and B viruses, thereby reducing the overall strain on testing facilities, especially during influenza season. Continued testing and surveillance of influenza viruses during the COVID-19 pandemic provide critical guidance on selection of candidate vaccine strains; these processes also identify antiviral resistance genes and novel influenza viruses that have pandemic potential (*8*).

We evaluated existing and novel SARS-CoV-2 primers and probes to identify the optimal SC2 assay components for this quadruplex rRT-PCR (Appendix Table 1). The SC2 assay components are selective for the 3′ region of the SARS-CoV-2 genome from the carboxy terminus of the nucleocapsid (N) gene into the 3′ untranslated region (UTR). The primer and probe sequences for the influenza A (InfA), influenza B (InfB), and RP targets are identical to those used in the singleplex assays of the US Food and Drug Administration (FDA)–approved CDC Human Influenza Virus Real-Time RT-PCR Detection and Characterization Panel [510(k) no. K200370] (*9*). The Flu SC2 Multiplex Assay is selective for the matrix (M) gene segment of the influenza A virus, the nonstructural (NS) gene segment of the influenza B virus, and the human ribonuclease P/MRP subunit P30 gene; the InfA assay is designed for universal detection of all influenza A viruses and InfB assay is designed for universal detection of all influenza B viruses (*10*–*14*). The InfA assay was recently updated to address evolutionary changes and reactivity challenges; the updated CDC Human Influenza Virus Real-Time RT-PCR Diagnostic Panel was cleared by FDA in 2020 (*9*). On July 2, 2020, FDA granted an emergency use authorization (EUA) for in vitro diagnostic use of the Flu SC2 Multiplex Assay (*15*).

**Table 1:** Primers and probes used in the Influenza SARS-CoV-2 Multiplex Assay*

Primer or probe	Oligonucleotide sequence, 5′→3′	Target gene or region	Nucleotide position†	Concentration, µM‡
InfA				
Forward primer 1	CAA GAC CAA TCY TGT CAC CTC TGAC	Matrix protein	143–167	3.33
Forward primer 2	CAA GAC CAA TYC TGT CAC CTY TGAC		143–167	3.33
Reverse primer 1	GCA TTY TGG ACA AAV CGT CTA CG		248–226	5
Reverse primer 2	GCA TTT TGG ATA AAG CGT CTA CG		248–226	1.67
InfB				
Forward primer	TCC TCA AYT CAC TCT TCG AGC G	Nonstructural protein	746–767	6.67
Reverse primer	CGG TGC TCT TGA CCA AAT TGG		848–828	6.67
RP				
Forward primer	AGA TTT GGA CCT GCG AGC G	Human RNase P	50–67	6.67
Reverse primer	GAG CGG CTG TCT CCA CAA GT		50–67	6.67
SARS-CoV-2				
Forward primer	CTG CAG ATT TGG ATG ATT TCT CC	Nucleoprotein–3′ untranslated region	29463–29485	6.67
Reverse primer	CCT TGT GTG GTC TGC ATG AGT TTA G		29554–29530	6.67
InfA probe§	TGC AGT CCT /ZEN/ CGC TCA CTG GGC ACG	Matrix protein	224–201	1.67
InfB probe¶	CCA ATT CGA /ZEN/ GCA GCT GAA ACT GCG GTG	Nonstructural protein	790–817	1.67
RP probe#	TTC TGA CCT /TAO/ GAA GGC TCT GCG CG	Human RNase P	71–93	1.67
SARS-CoV-2 probe**	ATT GCA ACA /TAO/ ATC CAT GAG CAG TGC TGA CTC	Nucleoprotein–3′ untranslated region	29491–29520	1.67

*Multiplex real-time reverse transcription PCR for influenza A virus, influenza B virus, and SARS-CoV-2. InfA, influenza A; InfB, influenza B; RP, RNase P; SARS-CoV-2, severe acute respiratory syndrome coronavirus 2. †Nucleotide positions are indicated as location in the coding domain sequences for the ribonuclease P/MRP subunit p30 (human; GenBank accession no. NM-004613), M gene (GISAID accession no. EPI 1312561) of A/Brisbane/02/2018-H1N1 (InfA), NS gene (GenBank accession no. CY232070) of B/Colorado/06/2017 (InfB), or Wuhan-Hu-1 (SARS-CoV-2; GenBank accession no. NC045512). ‡Multiplex assay is supplied with a tube of primers mixed in 1.5 mL aqueous solution and a tube of probes mixed in 1.5 mL aqueous solution. §Probe labeled with the reporter molecule 6-carboxyfluorescein at the 5′ end, a ZEN quencher between nt 9 and 10, and an Iowa Black FQ quencher at the 3′ end (Integrated DNA Technologies, Inc., https://www.idtdna.com). Probe and primer sequences are identical to InfA sequences used by the CDC Human Influenza Real-Time RT-PCR Diagnostic Panel [510(k) no. K200370] (*9*). ¶Probe labeled with Yakima Yellow reporter at the 5′ end, a ZEN quencher between nt 9 and 10, and an Iowa Black FQ quencher at the 3′ end (Integrated DNA Technologies, Inc.). Probe and primer sequences identical to InfB sequences used by the CDC Human Influenza Virus Real-Time RT-PCR Detection and Characterization Panel [510(k) no. K200370] (*9*). #Probe labeled with the CY5 reporter at the 5′ end, a TAO quencher between nt 9 and 10, and an Iowa Black RQ quencher at the 3′ end (Integrated DNA Technologies, Inc.). Probe and primer sequences identical to RP sequences in the CDC Human Influenza Virus Real-Time RT-PCR Detection and Characterization Panel [510(k) no. K200370]. **Probe labeled with the Texas Red-XN reporter at the 5′ end, a TAO quencher between nt 9 and 10, and an Iowa Black RQ quencher at the 3′ end (Integrated DNA Technologies, Inc.)

Multiplex detection of RNA from influenza A virus, influenza B virus, and SARS-CoV-2 can increase testing capacity and reduce use of reagents. The increased throughput can preserve staff resources and reduce turnaround time. The Flu SC2 Multiplex Assay and similar panels identify co-infections or alternative causes of influenza-like and COVID-19–like illnesses. The Flu SC2 Multiplex Assay can enable collection of critical data on influenza A and B viruses and SARS-CoV-2, as well as the prevalence of co-infection among these respiratory viruses.

## Materials and Methods

### Influenza Viruses and SARS-CoV-2

Influenza viruses were grown to high titer in Madin-Darby Canine Kidney cells or embryonated chicken eggs. Infectious virus titer in the cell culture supernatant or allantoic fluid was measured by using 50% tissue culture infectious dose (TCID_50_) or 50% egg infectious dose (EID_50_) (*16*). The SARS-CoV-2 virus (2019-nCoV/USA-WA1/2020; GenBank accession no. MT576563) was grown to high titer in Vero cells; the infectious virus titer in the cell culture supernatant was measured by using TCID_50_ (*16*). Total nucleic acids were extracted by using the EZ1 DSP Virus Kit on the EZ1 Advanced XL automated extractor (QIAGEN, https://www.qiagen.com).

### Primers and Probes

Primers and probes were selected from highly conserved regions of the SARS-CoV-2 genome based on ≈4,000 sequences available in GISAID (https://www.gisaid.org) in March 2020 (Table 1). Primer Express 3.0.1 software (Thermo Fisher Scientific, https://www.thermofisher.com) was used to design primers that had annealing temperatures of ≈60°C and probes that had annealing temperatures of ≈68°C.

The multiplex assay probes were synthesized by using ZEN or TAO Double-Quenched Probes labeled at the 5′ end using reporter 6-carboxyfluorescein (FAM) for InfA, Yakima Yellow for InfB, Texas Red-XN for SARS-CoV-2, and Cyanine 5 (Cy 5) for RP targets (Integrated DNA Technologies, Inc., https://www.idtdna.com). The InfA and InfB probes were quenched with ZEN between nucleotides 9 and 10 and with Iowa Black FQ at the 3′ end; the SARS-CoV-2 and RP probes were quenched with TAO between nucleotides 9 and 10 and with Iowa Black RQ at the 3′ end (Integrated DNA Technologies, Inc.). Primers and Taqman hydrolysis probes were synthesized by Integrated DNA Technologies and the CDC Biotechnology Core Facility Branch (Division of Scientific Resources, National Center for Emerging and Zoonotic Infectious Diseases; Atlanta, Georgia, USA).

### rRT-PCR Reaction Conditions

The rRT-PCR reactions of the Flu SC2 Multiplex Assay were optimized and conducted by using the TaqPath 1-Step Multiplex Master Mix (No Rox) (Thermo Fisher Scientific) and the 7500 Fast Dx Real-Time PCR Instrument (Thermo Fisher Scientific). The final volume of 25 μL included 6.25 μL of TaqPath 1-Step Multiplex Master Mix (No Rox) and 5 μL RNA. We used final concentrations of 400 nmol for the InfA F1 and F2 primers, 600 nmol for the InfA R1 primer, and 200 nmol for the InfA R2 primer; all other primers had final concentrations of 800 nmol. Probes had a final concentration of 200 nmol. Reaction conditions for the multiplex rRT-PCR were based on conditions for the CDC rRT-PCR Flu Panel, but we reduced the reverse transcription step from 30 min to 15 min (*9*,*17*). We used the following thermocycling conditions for rRT-PCR: 25°C for 2 min, 50°C for 15 min, *Taq* activation at 95°C for 2 min, 45 cycles at 95°C for 15 sec, and 55°C for 30 sec. We conducted comparator reactions using influenza singleplex rRT-PCR and the CDC 2019-nCoV Real-Time RT-PCR Diagnostic Panel, as described previously (*7*,*10*,*17*).

### Analytical Sensitivity and Specificity

A quantified synthetic RNA material (Armored RNA Quant CDC-9; Asuragen, Inc., https://asuragen.com) was used to test analytical sensitivity. The synthetic RNA included primer–probe region sequences derived from the M gene of A/Brisbane/02/2018_(H1N1)pdm09 (GISAID accession no. EPI1799928), for the InfA target, the NS gene of B/Colorado/06/2017_Victoria (GISAID accession no. EPI1056634) for the InfB target, and the *Homo sapiens* (human) ribonuclease P/MRP subunit P30 gene for the RP target. We used RNA extracted from propagated, A/Illinois/20/2018_(H1N1)pdm09 (GISAID accession no. EPI1220313; GenBank accession no. MH359945), and B/Colorado/06/2017_Victoria viruses to test the analytical sensitivity of the InfA and InfB targets. We used Twist Synthetic SARS-CoV-2 RNA Control 2 (Twist Bioscience, https://www.twistbioscience.com) and RNA extracted from propagated SARS-CoV-2 virus (2019-nCoV/USA-WA1/2020) to assess analytical sensitivity of the SARS-CoV-2 target.

We evaluated assay specificity by using a panel of influenza A virus, influenza B virus, and SARS-CoV-2. This panel included influenza A(H1/H3) variant viruses that usually circulate among swine and have caused outbreaks and pandemics in human populations (*18*–*22*).

We used a collection of influenza C viruses, coronaviruses, and human noninfluenza respiratory pathogens to test the analytical specificity of the Flu SC2 Multiplex Assay. We also tested the specificity of the SARS-CoV-2 target with an RNA transcript generated from a clone representing nt 27768–29738 of the severe acute respiratory syndrome coronavirus (SARS-CoV)/Urbani genome, which contains the entire N gene through the 3′-terminus, and a full SARS-CoV viral genome.

To test sensitivity to co-infection, we created a serial dilution with nucleic acids extracted from A/Illinois/20/2018_(H1N1)pdm09, B/Colorado/06/2017_Victoria, 2019-nCoV/USA-WA1/2020, and adenocarcinomic human alveolar basal epithelial cells (A549). We tested the dilution by the Flu SC2 Multiplex Assay, influenza A and influenza B singleplex rRT-PCR from the CDC rRT-PCR Flu Dx Panel Influenza A/B Typing Kit, and the N1 component of the CDC 2019-nCoV Real-Time RT-PCR Diagnostic Panel.

### In Silico Analysis

We tested the specificity and sensitivity of each primer and probe oligonucleotide sequence for the SARS-CoV-2 target of the Flu SC2 Multiplex Assay by BLAST analysis (https://blast.ncbi.nlm.nih.gov/blast.cgi) against the nr/nt database and the National Center for Biotechnology Iinformation and GISAID β Coronaviridae nucleotide database. We analyzed results and assessed for potential non–SARS-CoV-2 matches (Appendix). We compared the primer and probe sequences with SARS-CoV-2 variant sequences available in GISAID on January 19, 2021, including 501Y.V1, a B.1.1.7 variant from the United Kingdom; 501Y.V2, a B.1.351 variant from South Africa; and 501Y.V3, a P.1 variant from Brazil.

### Assay Performance with Clinical Specimens

We evaluated the clinical performance of the Flu SC2 Multiplex Assay using 104 upper and lower respiratory specimens, including oral swab, throat swab, nasopharyngeal swab, oropharyngeal swab, and sputum samples. Total nucleic acids were extracted from 120 μL of each clinical specimen by using the EZ1 DSP Virus Kit on the EZ1 Advanced XL automated extractor (QIAGEN). The extracted material was eluted in 120 μL elution buffer. Specimens were tested with the Flu SC2 Multiplex Assay, the CDC Human Influenza Real-Time RT-PCR Diagnostic Panel: Influenza A/B Typing Kit version 2 [510 (k) no. K200370], or the CDC 2019-nCoV Real-Time RT-PCR Diagnostic Panel, as described previously (*7*,*9*).

## Results

### Developing the SARS-CoV-2 Target

We identified candidate SARS-CoV-2 targets and evaluated them by an in silico screening process. This process identified targets with very few mismatches across the available SARS-CoV-2 genomes and accounted for RNA structural elements known to be essential for related betacoronaviruses. In total, we tested 17 SARS-CoV-2 assay designs in singleplex format; we subsequently tested a subset of these candidates using the multiplex format, including published targets in the RNA-dependent RNA polymerase and E gene regions (Appendix Table 1) (*23*). We selected for the assay the SARS-CoV-2 target with the highest levels of sensitivity and specificity and that accurately identified residual clinical respiratory specimens.

The SARS-CoV-2 assay is selective for the 3′ region of the SARS-CoV-2 genome from the carboxy terminus of the of the N gene into the 3′-UTR (Appendix Figure). This region is expressed at high levels in infected cells and is highly conserved because it encodes a cis-acting RNA pseudoknot essential for the transcription and replication of closely related betacoronaviruses (*24*).

### Analytical Sensitivity of Flu SC2 Multiplex Assay

We determined the analytical sensitivity of the Flu SC2 Multiplex Assay by calculating the limits of detection using extracted RNA from influenza A virus, influenza B virus, and SARS-CoV-2. We used serial 10-fold dilutions of extracted RNA to identify an endpoint for detection with each primer and probe set included in the multiplex assay (data not shown). After a detection range was established, we tested serial 5-fold dilutions of extracted RNA from each virus at titers near the limit of detection (LOD) with the Flu SC2 Multiplex Assay, the CDC 2019-nCoV Real-Time RT-PCR Diagnostic Panel, or the CDC rRT-PCR Flu Dx Panel: Influenza A/B Typing Kit version 2 [510 (k) no. K200370] (Table 2). We determined the limits of detection to be 10^2.0^ TCID_50_ for influenza A, 10^2.2^ EID_50_ for influenza B, and 10^0.3^ TCID_50_ for SARS-CoV-2 (Table 2). These values correspond to 10^–0.3^ TCID_50_ for each influenza A reaction, 10^–0.1^ EID_50_ for influenza B, and 10^–2.0^ TCID_50_ for SARS-CoV-2 (i.e., 5 µl RNA/reaction). We confirmed the LOD through further testing of 20 replicate viral isolates mixed with A549 cells at the established LOD and at a 5-fold dilution step above the established LOD; this process demonstrated that the multiplex assay can detect >95% of samples at the lowest detectable concentrations (Table 3; Appendix Table 2). The SD across the 20-replicate experiment was very low, demonstrating the consistency of the multiplex results even at the LOD (Table 3).

**Table 2:** Sensitivity of the Influenza SARS-CoV-2 Multiplex Assay compared with singleplex assays*

Viral titers†	Cycle threshold value‡						
	Multiplex				Singleplex		
A/Illinois/20/2018_(H1N1)pdm09						
10^4.1^	23.50	23.59	23.17		24.97	24.97	24.66
10^3.4^	26.57	26.50	26.81		27.71	27.49	27.50
10^2.7^	29.90	30.20	30.15		30.50	29.97	29.74
** 10^2.0^**	35.58	35.24	36.17		32.32	32.43	33.63
10^1.3^	42.23	37.28	0		36.01	34.61	34.96
B/Colorado/06/2017_Victoria							
10^4.3^	24.47	24.44	24.31		25.80	25.68	25.93
10^3.6^	27.57	27.45	27.71		28.84	28.98	29.30
10^2.9^	31.09	30.17	30.47		32.10	32.10	32.38
** 10^2.2^**	34.38	33.49	34.43		35.19	35.49	35.99
10^1.5^	39.75	0	0		0	0	0
2019-nCoV/USA-WA1/2020							
10^2.4^	25.41	25.8	25.42		26.48	26.57	26.46
10^1.7^	28.69	28.87	28.5		30.26	29.77	29.51
10^1.0^	31.31	31.42	31.32		32.74	33.17	32.27
** 10^0.3^**	35.14	36.36	34.58		36.10	35.34	35.81
10^–0.4^	0	0	0		37.16	0	0

*Multiplex PCR is selective for influenza A virus, influenza B virus, and SARS-CoV-2. Boldface type indicates limits of detection. SARS-CoV-2, severe acute respiratory syndrome coronavirus 2. †Viral titers are in relation to 50% tissue culture infectious dose, except for B/Colorado/06/2017, which is in relation to 50% egg infectious dose. ‡The multiplex cycle threshold values were derived from Influenza SARS-CoV-2 Multiplex Assay. The singleplex cycle threshold values were derived from the InfA (for influenza A), InfB (for influenza B), or N1 singleplex assays (for SARS-CoV-2). The N1 singleplex assay is a component of the CDC 2019-nCoV Real-Time RT-PCR Diagnostic Panel (*7*). The InfA and InfB singleplex assays are components of the CDC Human Influenza Virus Real-Time RT-PCR Detection and Characterization Panel [510(k) no. K200370] (*9*). Each assay was performed in triplicate.

**Table 3:** Confirmation of established limits of detection of the Influenza SARS-CoV-2 Multiplex Assay*

Viral titer†	Influenza A			Influenza B			SARS-CoV-2	
	No. (%) positive	Mean C_t_ ±SD		No. (%) positive	Mean C_t_ ±SD		No. (%) positive	Mean C_t_ ±SD
A/Illinois/20/2018_(H1N1)pdm09								
10^2.7^	20 (100)	29.71 ±0.51		0	NA		0	NA
** 10^2.0^**	20 (100)	33.55 ±1.15		0	NA		0	NA
B/Colorado/06/2017_Victoria								
10^2.9^	0	NA		20 (100)	29.80 ±0.74		0	NA
** 10^2.2^**	0	NA		20 (100)	32.70 ±0.48		0	NA
2019-nCoV/USA-WA1/2020								
10^1.0^	0	NA		0	NA		20 (100)	32.59 ±0.78
** 10^0.3^**	0	NA		0	NA		19 (95)	34.71 ±1.03

*Multiplex real-time reverse transcription PCR for influenza A virus, influenza B virus, and SARS-CoV-2. Boldface type indicates limits of detection. C_t_, cycle threshold; NA, not applicable; SARS-CoV-2, severe acute respiratory syndrome coronavirus 2; SD, standard deviation. †Viral titers are in relation to 50% tissue culture infectious dose, except for B/Colorado/06/2017, which is in relation to 50% egg infectious dose.

We used an engineered RNA construct (Armored RNA Quant CDC-9; Asuragen, Inc.) containing the target sequences for the InfA, InfB, and RP assays to test the copy number sensitivity of the multiplex assay through serial dilutions. We assessed copy number sensitivity of the SARS-CoV-2 assay by using a serial dilution of a synthetic SARS-CoV-2 genome (GenBank accession no. MN908947.3; Twist Bioscience). All targets in the assay could detect as few as 5 RNA copies per reaction (Table 4).

**Table 4:** Evaluation of the Influenza SARS-CoV-2 Multiplex Assay sensitivity using quantified synthetic RNAs*

RNA copies/reaction	Cycle threshold values														
	Influenza A				Influenza B				SARS-CoV-2				RNase P (human)		
50,000	23.72	24.02	23.86	22.02	21.59	21.88	20.05	20.26	20.08	22.02	22.13	21.69
5,000	27.01	27.38	28.01		25.17	25.03	25.04		24.03	24.12	24.15		25.12	24.68	25.09
500	31.74	31.73	32.48		28.52	28.02	28.88		27.25	28.01	27.60		28.19	28.02	28.11
50	35.60	35.34	36.65		32.58	31.58	31.17		31.89	31.33	32.93		31.47	31.54	30.59
5	36.50	38.40	0		33.76	34.65	36.15		34.24	34.03	34.25		34.33	35.42	39.02

*Multiplex real-time reverse transcription PCR for influenza A virus, influenza B virus, and SARS-CoV-2. Influenza A, Influenza B, and RP targets were quantified using Armored RNA Quant CDC-9 (Asuragen, Inc., https://asuragen.com), which includes the M gene of influenza A virus strain A/Brisbane/02/2018_H1N1, the NS gene of influenza B virus strain B/Colorado/06/2017, and RP; the SARS-CoV-2 target was quantified using Twist Synthetic SARS-CoV-2 RNA (Twist Bioscience, https://www.twistbioscience.com). SARS-CoV-2, severe acute respiratory syndrome coronavirus 2.

### Analytical Specificity of Flu SC2 Multiplex Assay

Initially, the Flu SC2 Multiplex Assay was screened using no template control reactions; we found no intramolecular or intermolecular nonspecific interactions that resulted in any products (data not shown). The specificity of the primers and probes was evaluated with viral RNA from 13 influenza A, 2 influenza B, and 1 SARS-CoV-2 isolate. The viral RNAs were tested at high and low titers; each assay accurately detected the corresponding viral target (Table 5). We observed no cross-reactivity among the 4 targets within the assays, nor did we observe any bleed-through fluorescence imaging from neighboring channels when testing the individual assays (Table 5).

**Table 5:** Evaluation of the Influenza SARS-CoV-2 Multiplex Assay specificity*

Virus strain	Lineage	GISAID accession no.	Con†	Cycle threshold value											
				Influenza A				Influenza B				SARS-CoV-2			
Influenza A												
A/Florida/81/2018	A(H1N1) pdm09	EPI1310819	10^8.1^	13.97	14.00	14.02		0	0	0		0	0	0
			10^3.1^	27.48	28.16	27.74		0	0	0		0	0	0
A/Kansas/14/2017	A(H3N2)	EPI1653963	10^8.5^	13.62	13.66	13.68		0	0	0		0	0	0
			10^5.5^	25.07	25.00	25.01		0	0	0		0	0	0
A/Ohio/35/2017	A(H1N2)v	EPI1056728	10^6.9^	14.71	14.90	14.84		0	0	0		0	0	0
			10^1.9^	30.91	31.25	30.99		0	0	0		0	0	0
A/chicken/Pennsyl- vania/298101- 4/2004	A(H2N2)	EPI229365	10^9.5^	15.60	15.66	15.74		0	0	0		0	0	0
			10^3.5^	33.40	33.20	34.71		0	0	0		0	0	0
A/Ohio/13/2017	A(H3N2)v	EPI1056648	10^6.6^	20.85	20.96	20.86		0	0	0		0	0	0
			10^1.6^	35.48	35.49	33.98		0	0	0		0	0	0
A/canine/Florida/ 43/2004	A(H3N2)		10^8.1^	19.61	19.69	19.44		0	0	0		0	0	0
		EPI98471	10^4.1^	34.10	35.45	36.87		0	0	0		0	0	0
A/equine/Ohio/01/ 2003	A(H3N8)	DQ124188§	10^8.4^	16.50	16.70	16.68		0	0	0		0	0	0
			10^3.4^	31.01	31.55	31.07		0	0	0		0	0	0
A/Northern pintail/Washington /40964/2014	A(H5N2)	EPI860995	10^9.4^	16.39	16.43	16.49		0	0	0		0	0	0
			10^4.4^	36.89	36.45	34.43		0	0	0		0	0	0
A/gyrfalcon/Wash- ington/41088- 6/2014	A(H5N8)	EPI569393	10^9.75^	14.12	14.10	14.13		0	0	0		0	0	0
			10^4.75^	29.60	29.38	29.90		0	0	0		0	0	0
A/chicken/Califor- nia/32213–1/2000	A(H6N2)	EPI1915583	10^9.2^	14.97	15.05	15.08		0	0	0		0	0	0
			10^3.2^	34.19	32.52	32.49		0	0	0		0	0	0
A/feline/New York/16-040082- 1/2016	A(H7N2)	EPI985440	10^10.2^	15.76	15.94	16.00		0	0	0		0	0	0
			10^5.2^	28.16	28.30	28.46		0	0	0		0	0	0
A/Taiwan/1/2017	A(H7N9)	EPI917065	10^9.5^	16.69	16.90	16.99		0	0	0		0	0	0
			10^3.5^	32.20	33.11	32.53		0	0	0		0	0	0
A/Bangladesh/ 0994/2011	A(H9N2)	EPI445991	10^10.5^	18.03	18.14	18.21		0	0	0		0	0	0
			10^4.5^	34.25	35.32	36.90		0	0	0		0	0	0
Influenza B														
B/Maryland/15/ 2016	B(Victoria)	EPI1255266	10^8.5^	0	0	0		13.46	13.49	13.47		0	0	0
			10^2.5^	0	0	0		30.82	31.07	31.20		0	0	0
B/Phuket/3073/ 2013	B (Yamagata)	EPI1799818	10^8.9^	0	0	0		13.66	13.67	13.68		0	0	0
			10^2.9^	0	0	0		31.87	31.77	32.18		0	0	0
SARS-CoV-2														
2019-nCoV/USA- WA1/2020	Beta-coronavirus	MT 576563	10^4.5^	0	0	0		0	0	0		18.34	18.55	18.41
			10^0.5^	0	0	0		0	0	0		33.11	34.15	34.88

*Multiplex real-time reverse transcription PCR for influenza A virus, influenza B virus, and SARS-CoV-2. Evaluation conducted in triplicate. Con, concentration; SARS-CoV-2, severe acute respiratory syndrome coronavirus 2. ‡Concentration in relation to 50% tissue culture infectious dose. §GenBank accession number.

To confirm that the SARS-CoV-2 assay was specific to that virus, we tested 6 known human coronaviruses, 2 alphacoronaviruses, 2 group A betacoronaviruses, and 2 group B betacoronavirus (i.e., SARS-CoV and Middle East respiratory syndrome coronavirus [MERS-CoV]), as well as an RNA transcript including the entire SARS-CoV N gene region through the 3′ UTR. No cross-reactivity was observed, demonstrating the high specificity of the assay (Appendix Table 3).

To further evaluate the specificity of the multiplex assay, we also tested common respiratory pathogens and genetic near neighbors of viruses selected for by the assay. Nucleic acids from high titer viral preparations were extracted and tested with the Flu SC2 Multiplex Assay; no cross-reactivity was observed (Appendix Table 4).

An extensive in silico BLAST analysis of the primer and probe sequences for the SARS-CoV-2 target confirmed that the assay is specific to SARS-CoV-2; no evidence of non–SARS-CoV-2 target matches was found (Figure; Appendix Table 5). These results demonstrate that Flu SC2 Multiplex Assay is specific to influenza A viruses, influenza B viruses, and SARS-CoV-2; it does not detect other respiratory pathogens or close relatives, including SARS-CoV.

**Figure:**
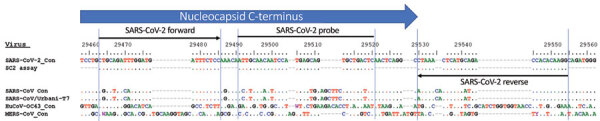
Alignment of SARS-CoV-2-specific PCR with consensus sequences for SARS-CoV-2, SARS-CoV, MERS-CoV, and HuCoV-OC43. Consensus sequence for SARS-CoV/Urbani-T7 was reverse transcribed from SARS-CoV strain Urbani (GenBank accession no. AY278741). HuCoV-OC43, human coronavirus OC43 consensus sequence; MERS-CoV, Middle East respiratory syndrome coronavirus; SARS-CoV, severe acute respiratory syndrome coronavirus; SARS-CoV-2, severe acute respiratory syndrome coronavirus 2.

An in silico analysis compared the genomes of the emerging SARS-CoV-2 variants B.1.1.7, B.1.351, and P.1 with the sequence of the SARS-CoV-2 target. This analysis demonstrated that during January 2021, most (>99.5%) of the variant virus sequencing data was identical to the SARS-CoV-2 target sequence; of the genomes that had <100% match, none except 2 sequences displayed >1 mismatch for any region of the assay (Appendix Table 6). Therefore, the Flu SC2 Multiplex Assay should accurately detect the B.1.1.7, B.1.351, and P.1 SARS-CoV-2 variants.

### Co-Infection Sensitivity of Flu SC2 Multiplex Assay

We evaluated the analytical sensitivity of the multiplex assay in the context of a mock co-infection scenario by testing a mixture of nucleic acids extracted from influenza A, influenza B, SARS-CoV-2, and A549 cells with the Flu SC2 Multiplex Assay and the InfA, InfB, and N1 singleplex assays (*7*,*9*). The results demonstrated that the multiplex assay can detect all 4 targets simultaneously at comparable or higher sensitivity levels than each singleplex comparator (Appendix Table 7).

### Performance on Clinical Specimens

We evaluated the clinical performance of the multiplex assay by using residual clinical respiratory specimens. Nucleic acids were extracted from 104 prospective and retrospective clinical specimens, including 33 SARS-CoV-2–positive, 30 influenza A–positive, 30 influenza B–positive, and 11 negative residual clinical samples. The samples were tested with the Flu SC2 Multiplex Assay; the results were in 100% agreement with the expected value for each specimen (Table 6; Appendix Tables 8, 9).

**Table 6:** Clinical performance of the Influenza SARS-CoV-2 Multiplex Assay*

Specimen type (no.)	Influenza A–positive	Influenza B–positive	SARS-CoV-2–positive	Negative for all 3 viral targets
Influenza A (30)	30	0	0	0
Influenza B (30)	0	30	0	0
SARS-CoV-2 (33)	0	0	33	0
Negative for all 3 viral targets (11)	0	0	0	11

*Multiplex real-time reverse transcription PCR for influenza A virus, influenza B virus, and SARS-CoV-2. SARS-CoV-2, severe acute respiratory syndrome coronavirus 2; (+), positive.

## Discussion

SARS-CoV-2 emerged in December 2019 and quickly spread, causing the COVID-19 pandemic. As the SARS-CoV-2 infection rate increased, the demand for viral diagnostic testing also increased. The Flu SC2 Multiplex Assay increases throughput and uses less reagent than the CDC 2019-nCoV Real-Time RT-PCR Diagnostic Panel, thus improving SARS-CoV-2 testing efficiency. The multiplex assay enables laboratories to simultaneously test for influenza viruses and SARS-CoV-2, an application that is especially useful because influenza virus and SARS-CoV-2 infections cause similar signs and symptoms (*25*,*26*). Although not described in this article, additional enzyme master mix combinations, nucleic acid extraction platforms, and an alternative manufacturer were added to the assay EUA, further improving its utility (*15*,*27*). CDC granted the right of reference to all data submitted to the FDA for EUA authorization of the Flu SC2 Multiplex Assay. Several commercial providers have leveraged the data to produce multiplex kits, including the BioSearch Valuepanel (LGC BioSearch Technologies, https://www.biosearchtech.com), PrimeTime SARS-CoV-2/Flu Test Integrated DNA Technologies, Inc., Accuplex (includes assay for human respiratory syncytial virus) (SeraCare Life Sciences, Inc., https://www.seracare.com), BioRad Reliance (Bio-Rad Laboratories, Inc., https://www.bio-rad.com), and FLU SC2 RT-PCR (InGenuityD Diagnostics, https://ingenuityd.com).

The analytical sensitivity of the Flu SC2 Multiplex Assay was evaluated; each component was comparable to the singleplex versions of each assay. The assay detects titers as low as 10^2.2^–10^0.3^ TCID_50_ or EID_50_ (or 10^–2.0^–10^−0.1^ TCID_50_ or EID_50_/reaction) of influenza A viruses, influenza B viruses, and SARS-CoV-2, or as few as 5 RNA copies/reaction. We observed no cross-reactivity among the targets, even at high viral titers; none with the other 6 known human coronaviruses, including SARS-CoV and MERS-CoV; and none with influenza C cultured viruses or other common noninfluenza respiratory pathogens (*28*). The Flu SC2 assays manufactured by CDC are evaluated to ensure that the LOD of each lot is comparable with the LOD established in the EUA. Quality assessments ensure limited variability: lots that have a variance of >2 cycle thresholds from the EUA submission data against standard quality control virus dilution series are deemed unacceptable for distribution (data not shown). These standards ensure that sensitivity and specificity are maintained through the manufacturing process.

The SARS-CoV-2 target used by the multiplex panel was selected from a conserved and vital region of the N gene (*29*). Analytical evaluation and in silico analysis demonstrated the target is sensitive and specific to SARS-CoV-2 and will not detect other human coronaviruses, including SARS-CoV and MERS-CoV. The in silico analysis of 376,469 SARS-CoV-2 sequences available in GISAID in January 2021 indicated that >99.9% of the viruses have <1 mismatch within a single primer or probe of the SARS-CoV-2 assay (Appendix Table 5). An in silico analysis of genomes from the emerging SARS-CoV-2 B.1.1.7, B.1.351, and P.1 variants demonstrated that the target is identical to the genome sequence for >99.5% of these variant genomes (Appendix Table 6). The Flu SC2 Multiplex Assay should detect these emerging variants because the mutations associated with these variants are located within a different region of the genome than the target.

The Flu SC2 Multiplex Assay was evaluated using a reference panel developed by the FDA for assessing diagnostic nucleic acid amplification tests for SARS-CoV-2 (*30*,*31*). The panel consisted of reference SARS-CoV-2 material, blinded samples, and a protocol provided by the FDA. The evaluation included range finding and confirmatory studies for LOD, as well as blinded sample testing to establish specificity and further confirmation of the LODs. The LOD of the Flu SC2 Multiplex Assay using the FDA panel was 5.7 × 10^3^ nucleic acid amplification test detectable units/mL, with no observable cross-reactivity with MERS-CoV (*32*).

In summary, the Flu SC2 Multiplex Assay demonstrates a high level of specificity and sensitivity. In a single reaction, it can detect and distinguish 3 major respiratory viruses as well as the human quality control target, thereby increasing the testing throughput. Additional advantages of the Flu SC2 Multiplex Assay include fewer freeze-thaw cycles, decreased potential for contamination through a reduction in the number of reactions, and fewer opportunities for pipetting errors. With this multiplex assay, users can rapidly test large amounts of samples. Although the influenza season for 2020–21 had historically few cases, this assay will be beneficial in upcoming influenza seasons when influenza might co-circulate with SARS-CoV-2.

Appendix Additional information on multiplex real-time RT-PCR for influenza A virus, influenza B virus, and SARS-CoV-2.
