# Supplementary material for: Multiplex Real-Time Reverse Transcription PCR for Influenza A Virus, Influenza B Virus, and Severe Acute Respiratory Syndrome Coronavirus 2
Source: Emerg Infect Dis. 2021 Jul;27(7):1821–30. doi: 10.3201/eid2707.210462 (PMC8237866; doi:10.3201/eid2707.210462)
Supplement: Appendix — Additional information on multiplex real-time RT-PCR for influenza A virus, influenza B virus, and SARS-CoV-2. [file 21-0462-Techapp-s1.pdf]

Article DOI: https://doi.org/10.3201/eid2707.210462

Multiplex Real-Time RT-PCR for Influenza A Virus, Influenza B Virus, and Severe Acute Respiratory Syndrome Coronavirus 2

Appendix

Appendix Methods

Severe Acute Respiratory Syndrome Coronavirus 2 In silico Analysis

The inclusivity/exclusivity of each primer and probe oligonucleotide sequence for the severe acute respiratory syndrome coronavirus 2 (SARS-CoV-2) target of the Flu SC2 Multiplex Assay was tested by using BLAST (http://blast.ncbi.nlm.nih.gov/Blast.cgi) against the nr/nt database. The database search parameters were as follows: the nucleotide collection consists of GenBank+EMBL+DDBJ+PDB+RefSeq sequences, but excludes expressed sequence tags, sequence tagged sites, genome survey sequences, whole-genome shotguns, transcription shotgun assemblies, and patent sequences, as well as phase 0, 1, and 2 high throughput genomic sequences and sequences >100 Mb; the database is nonredundant (identical sequences have been merged, although preserving the accession no., GenInfo identifier, title, and taxonomy information for each entry); the database was updated on January 12, 2021 (n = 66,364,420 sequences); the search parameters automatically adjust for short input sequences and the expected threshold is 1,000; the match score is 1 and mismatch score is −3; the penalty to create a gap is 5 and to extend a gap in an alignment is 2.

In addition, we tested each primer sequence against the NCBI β Coronaviridae nucleotide database (www.ncbi.nlm.nih.gov/genbank) to test for nonspecific hits within a targeted search space. The database search parameters were the same as described above with the following differences: the nucleotide collection consists of only betacoronavirus nucleotide sequence data; the database is nonredundant (identical sequences have been merged while preserving the accession, GI, title, and taxonomy information for each entry); the database was updated on January 19, 2021 (n = 58,646 sequences). We analyzed and assessed results for potential non–

SARS-CoV-2 target matches. We categorized results as perfect matches to the primer/probe sequences, close matches (≤2 nt edit distance), or distant matches (>2 nt edit distance). Results confirmed only direct matches to SARS-CoV-2 and immediate SARS-CoV-2 ancestors (i.e., no genomes identified with >2 nt mismatches).

Appendix Table 1. Additional primers and probes evaluated for the Influenza SARS-CoV-2 Multiplex Assay*

Assay	Description†	Oligonucleotide sequence (5′→3′)‡	Target gene	Nucleotide position§
N gene	Forward primer	CATTGGCATGGAAGTCACACCT	Nucleocapsid protein	29,230–29,251
	Probe	CGTGGTTGACCTACACAGSTGCCATCAA		29,259–29,286
	Reverse primer	CTTTGAAATTTGGATCTTTGTCATCC		29,315–29,290
E gene	Forward primer	CCTATTTCTGAACATGACTACCAGATTG	Envelope protein	25,924–25,951
	Probe	CACAGTCTTTTACTCCAGATTCCCATTTTTCAG		25,993–25,961
	Reverse primer	GTCTGAAGTGAAGTAACTGTGTAATACA		26,022–25,995
Coman's N gene	E_Sarbeco_F	ACAGGTACGTTAATAGTTAATAGCGT	Nucleocapsid protein	26,269–26,294
	E_Sarbeco_P1	ACACTAGCCATCCTTACTGCGCTTCG		26,332–26,357
	E_Sarbeco_R	ATATTGCAGCAGTACGCACACA		26,381–26,360
Coman's RdRp gene	RdRp_SARSr-F	GTGARATGGTCATGTGTGGCGG	RNA-dependent RNA polymerase	15,431–15,452
	RdRp_SARSr-P2	CAGGTGGAACCTCATCAGGAGATGC		15,470–15,494
	RdRp_SARSr-R	CARATGTTAAASACACTATTAGCATA		15,530–15,505

*Multiplex real-time reverse transcription PCR for influenza A virus, influenza B virus, and SARS-C0V-2. SARS-CoV-2, severe acute respiratory syndrome coronavirus 2.
†All probes labeled with the reporter dye Texas Red at the 5′-end and a Blackhole Quencher 2 at the 3′-end (LGC Biosearch Technologies, https://www.biosearchtech.com).
‡R indicates G/A single nucleotide polymorphism; S indicates G/C single nucleotide polymorphism.
§Nucleotide positions are indicated as location in the coding domain sequences of severe acute respiratory syndrome coronavirus 2 (Wuhan-Hu-1; GenBank accession no. NC045512).

Appendix Table 2. Confirmation of limits of detection of the Influenza SARS-CoV-2 Multiplex Assay*

Virus strain, titer*		Cycle threshold values															
		Influenza A				Influenza B				SARS-CoV-2				RNase P (human)			
A/Illinois/20/2018_(H1N1)pdm09																	
	$10^{2.7}$	29.83	30.15	29.22	29.84	0	0	0	0	0	0	0	0	26.92	27.03	26.90	26.62
		29.98	29.15	30.35	30.00	0	0	0	0	0	0	0	0	26.97	26.26	27.33	26.47
		29.59	29.86	29.47	31.05	0	0	0	0	0	0	0	0	26.92	26.87	26.30	27.25
		29.24	29.74	29.70	29.45	0	0	0	0	0	0	0	0	26.82	26.61	26.47	26.43
		29.86	28.51	29.81	29.48	0	0	0	0	0	0	0	0	26.72	26.76	26.58	26.48
	**$10^{2.0}$**	32.22	36.07	33.43	31.90	0	0	0	0	0	0	0	0	26.84	26.44	26.95	26.59
		32.80	34.74	32.89	33.42	0	0	0	0	0	0	0	0	26.28	26.36	26.65	26.74
		33.48	32.29	33.14	35.86	0	0	0	0	0	0	0	0	26.56	26.31	26.74	26.18
		32.81	33.99	35.33	34.00	0	0	0	0	0	0	0	0	26.60	26.47	26.52	26.76
		33.04	32.84	33.32	33.51	0	0	0	0	0	0	0	0	26.38	26.66	26.69	26.80
B/Colorado/06/2017_Victoria																	
	$10^{2.9}$	0	0	0	0	29.66	29.55	29.51	29.42	0	0	0	0	27.93	27.73	27.79	27.64
		0	0	0	0	29.52	29.63	29.63	29.46	0	0	0	0	27.73	28.13	27.84	27.51
		0	0	0	0	29.90	29.54	29.88	29.58	0	0	0	0	27.91	27.98	27.72	27.69
		0	0	0	0	29.39	29.55	29.74	29.31	0	0	0	0	27.46	27.8	27.58	27.53
		0	0	0	0	29.36	29.38	29.82	29.79	0	0	0	0	27.8	27.55	27.51	27.58
	**$10^{2.2}$**	0	0	0	0	32.43	33.35	33.51	32.21	0	0	0	0	27.73	27.96	27.75	27.66
		0	0	0	0	32.81	33.04	31.95	32.65	0	0	0	0	27.73	28.06	27.76	27.54
		0	0	0	0	32.12	32.47	32.66	33.30	0	0	0	0	27.65	27.57	27.82	27.84
		0	0	0	0	32.48	32.99	32.57	32.75	0	0	0	0	27.81	27.74	27.7	27.72
		0	0	0	0	32.52	33.70	32.42	32.10	0	0	0	0	27.58	27.58	27.84	27.7
2019-nCoV/USA-WA1/2020																	
	$10^{1.0}$	0	0	0	0	0	0	0	0	32.15	35.05	32.48	32.43	28.55	28.49	28.51	28.33
		0	0	0	0	0	0	0	0	31.68	33.02	32.85	32.55	28.33	28.52	28.26	28.48
		0	0	0	0	0	0	0	0	31.74	33.34	33.33	31.81	28.63	28.84	28.34	28.28
		0	0	0	0	0	0	0	0	32.24	32.71	32.27	31.90	28.36	28.53	28.43	28.29
		0	0	0	0	0	0	0	0	33.32	31.79	32.74	32.46	28.47	28.53	28.62	28.44
	**$10^{0.3}$**	0	0	0	0	0	0	0	0	0	34.20	34.46	34.16	28.16	28.44	28.68	28.55
		0	0	0	0	0	0	0	0	35.09	33.35	35.44	33.35	28.43	28.65	28.72	28.33
		0	0	0	0	0	0	0	0	34.94	36.59	36.13	34.11	28.14	28.37	28.48	28.29
		0	0	0	0	0	0	0	0	36.28	34.08	33.17	33.4	28.40	28.68	28.32	28.33
		0	0	0	0	0	0	0	0	34.16	35.73	35.30	34.72	28.40	28.27	28.25	28.56

*Multiplex real-time reverse transcription PCR for influenza A virus, influenza B virus, and SARS-CoV-2. SARS-CoV-2, severe acute respiratory syndrome coronavirus 2.
†Viral titers are in relation to 50% tissue culture infectious dose, except for B/Colorado/06/2017 B/ Victoria, which is in relation to 50% egg infectious dose. Bolded values indicate limits of detection.

Appendix Table 3. Coronavirus specificity evaluation of the Influenza SARS-CoV-2 Multiplex Assay*

				Cycle threshold value								
		GenBank	Infectious titers,	Influenza A			Influenza B			SARS-CoV-2		
Coronavirus	Genera	accession no.	ng/μL									
HCoV 229E	Alphacoronavirus	KU291448	6.2	0	0	0	0	0	0	0	0	0
HCoV NL63	Alphacoronavirus	KY862074	2.75	0	0	0	0	0	0	0	0	0
HCoV HKU1	Group A betacoronavirus	MH940245	3.23	0	0	0	0	0	0	0	0	0
HCoV OC43	Group A betacoronavirus	KU131570	1.05	0	0	0	0	0	0	0	0	0
MERS-CoV	Group B betacoronavirus	NC_019843	5.4	0	0	0	0	0	0	0	0	0
SARS-CoV	Group B betacoronavirus	EU034725	ND	0	0	0	0	0	0	0	0	0
SARS CoV†	Strain Urbani	AY278741	NA	0	0	0	0	0	0	0	0	0

*Multiplex real-time reverse transcription PCR for influenza A virus, influenza B virus, and SARS-CoV-2. NA, not applicable; ND, not determined; SARS-CoV-2, severe acute respiratory syndrome coronavirus 2.
†RNA transcript.

Appendix Table 4. Analytical specificity of the Influenza SARS-CoV-2 Multiplex Assay*

		GenBank		Cycle threshold values		
Pathogen	Strain	accession no.	Titer†	Influenza A	Influenza B	SARS-CoV-2
Viruses						
Influenza C	C/Minnesota/1/2016	EPI1033841	NA	0	0	0
	C/Minnesota/4/2015	EPI1033781	NA	0	0	0
	C/Minnesota/29/2015	EPI1033830	NA	0	0	0
Human adenovirus, type 1	Ad.71	EU934336	$10^{9.2}$	0	0	0
Human adenovirus, type 7a	S-1058	AY921616	$10^{7.1}$	0	0	0
Human parainfluenza 1	NA		3.0‡	0	0	0
Human parainfluenza 2	Greer	KT898924	$10^{3.1}$	0	0	0
Human parainfluenza 3	C-243	EU346887	$10^{7.9}$	0	0	0
Respiratory syncytial virus	CH93–18b	AF065252	$10^{6.8}$	0	0	0
Human rhinovirus A	1A		$10^{5.8}$	0	0	0
Enterovirus	Echo 6		$10^{6.9}$	0	0	0
Herpes simplex virus	KOS		$10^{8.4}$	0	0	0
Varicella zoster virus	AV92–3:H		$10^{4.4}$	0	0	0
Epstein-Barr virus	B95–8		1.7‡	0	0	0
Measles morbillivirus	Edmonston		$10^{5.2}$	0	0	0
Mumps virus	Enders		$10^{7.2}$	0	0	0
Cytomegalovirus	AD-169		$10^{6.9}$	0	0	0
Bacteria						
Bordetella pertussis	Tohama I		$10^{10.0}$§	0	0	0
Chlamydia pneumoniae	CM-1		40¶	0	0	0
Corynebacterium diphtheriae	NCTC 13129		57.4‡	0	0	0
Escherichia coli	K12		$10^{9.6}$§	0	0	0
Streptococcus pyogenes	7790–06		$10^{7.5}$§	0	0	0
Haemophilus influenzae	M15709		$10^{6.4}$§	0	0	0
Lactobacillus plantarum	NA		$10^{8.8}$§	0	0	0
Legionella pneumophila	Philadelphia-1		$10^{8.4}$§	0	0	0
Moraxella catarrhalis	M15757		$10^{9.5}$§	0	0	0
Mycobacterium tuberculosis	H37Ra		$10^{5.0}$§	0	0	0
Mycoplasma pneumoniae	PI 1428		$10^{9.0}$§	0	0	0
Neisseria elongata	NA		$10^{5.0}$§	0	0	0
Neisseria meningitidis	M2578		$10^{7.9}$§	0	0	0
Pseudomonas aeruginosa	NA		$10^{10.5}$§	0	0	0
Staphylococcus aureus	NA		$10^{10.7}$§	0	0	0
Staphylococcus epidermidis	NA		$10^{10.5}$§	0	0	0
Streptococcus pneumoniae	249–06		$10^{6.6}$§	0	0	0
Streptococcus salivarius	DSM 13084		109‡	0	0	0
Yeast						
Candida albicans	3147		$10^{8.5}$§	0	0	0

*Multiplex real-time reverse transcription PCR for influenza A virus, influenza B virus, and SARS-CoV-2. NA, not available; SARS-CoV-2, severe acute respiratory syndrome coronavirus 2.
†Values are in relation to 50% tissue culture infectious dose, except as indicated.
‡ng/μL.
§CFU/mL.
¶Infectious units/mL.

Appendix Table 5. In silico analysis of the primers and probe for severe acute respiratory syndrome coronavirus 2 in the Influenza SARS-CoV-2 Multiplex Assay*

	No. sequences (%)				
	Nucleotide mismatch				
Component	0	1	2	≥3	Total
Forward primer	404,998 (98.23)	7,209 (1.75)	75 (0.02)	11 (<0.01)	412,293 (100)
Probe	409,225 (99.26)	3,054 (0.74)	9 (<0.01)	4 (<0.01)	412,293 (100)
Reverse primer	405,169 (98.27)	7,091 (1.72)	25 (0.01)	8 (<0.01)	412,293 (100)

*Multiplex real-time reverse transcription PCR for influenza A virus, influenza B virus, and SARS-CoV-2. SARS-CoV-2, severe acute respiratory syndrome coronavirus 2.

Appendix Table 6. In silico analysis of the primers and probe for severe acute respiratory syndrome coronavirus 2 in the Influenza SARS-CoV-2 Multiplex Assay, compared with variants*

	No. sequences (%)				
	Nucleotide mismatch				
Component	0	1	2	≥3	Total
Forward primer	19,822 (99.85)	29 (0.15)	2 (0.01)	0	19,851 (100.00)
Probe	19,787 (99.68)	64 (0.32)	0	0	19,851 (100.00)
Reverse primer	19,805 (99.77)	46 (0.23)	0	0	19,851 (100.00)

*Multiplex real-time reverse transcription PCR for influenza A virus, influenza B virus, and SARS-CoV-2. Studied strains comprise B.1.1.7, B.1.351, and P.1 variants of SARS-CoV-2. SARS-CoV-2, severe acute respiratory syndrome coronavirus 2.

Appendix Table 7. Evaluation of limits of detection using simulated co-infection in the Influenza SARS-CoV-2 Multiplex Assay*

Viral titers (InfA/InfB/SARS-CoV-2)†	Cycle threshold values																	
	Multiplex									Singleplex								
	InfA			InfB			SARS-CoV-2			InfA			InfB			N1‡		
$10^{4.1}/10^{4.3}/10^{1.7}$	24.73	25.04	24.84	22.59	22.60	22.82	26.50	26.90	27.18	25.27	25.85	25.98	24.72	24.72	25.11	26.20	26.49	26.09
$10^{3.4}/10^{3.6}/10^{1.0}$	27.56	26.87	27.55	25.38	25.09	25.15	30.12	29.16	29.61	28.69	28.62	29.14	28.19	28.36	28.43	29.29	29.95	30.73
$10^{2.7}/10^{2.9}/10^{0.3}$	30.13	31.14	29.82	27.59	26.71	27.42	31.88	30.96	31.17	32.38	31.31	31.80	31.18	31.53	31.14	30.46	32.30	31.76
$10^{2.0}/10^{2.2}/10^{-0.4}$	35.32	33.30	33.01	30.35	30.40	30.72	34.17	35.03	0	34.47	34.68	35.14	34.72	34.16	36.94	34.16	0	0
$10^{1.3}/10^{1.5}/10^{-1.1}$	34.30	36.55	0	32.80	32.16	32.17	34.58	0	0	0	0	37.58	36.80	38.56	0	0	0	0

*Multiplex real-time reverse transcription PCR for influenza A virus, influenza B virus, and SARS-CoV-2. Evaluation conducted in triplicate. Inf A, influenza A; InfB, influenza B; SARS-CoV-2, severe acute respiratory syndrome coronavirus 2.

†Viral concentrations in relation to 50% tissue culture infectious dose (A/Illinois/20/2018_H1N1pdm09 or 2019-nCoV/USA-WA1/2020) or 50% egg infectious dose (B/Colorado/06/2017_Victoria)

‡CDC 2019-nCoV Real-Time RT-PCR Diagnostic Panel (*7*). The InfA and InfB singleplex assays are components of the CDC Human Influenza Virus Real-Time RT-PCR Detection and Characterization Panel [510(k) no. K200370] (*9*).

Appendix Table 8. Comparison of the Influenza SARS-CoV-2 Multiplex Assay and singleplex assays using clinical influenza specimens*

		Cycle threshold values				
		Multiplex			Singleplex	
Sample ID	Lineage	Influenza A	Influenza B	SARS-CoV-2	Influenza A	Influenza B
Influenza A						
InfA_1	A(H1N1)pdm09	22.40	0	0	22.32	0
InfA_2	A(H1N1)pdm09	31.31	0	0	30.04	0
InfA_3	A(H1N1)pdm09	26.70	0	0	25.96	0
InfA_4	A(H1N1)pdm09	29.48	0	0	28.93	0
InfA_5	A(H1N1)pdm09	27.64	0	0	27.08	0
InfA_6	A(H1N1)pdm09	25.42	0	0	24.94	0
InfA_7	A(H1N1)pdm09	25.71	0	0	25.97	0
InfA_8	A(H1N1)pdm09	29.35	0	0	29.32	0
InfA_9	A(H1N1)pdm09	28.13	0	0	27.82	0
InfA_10	A(H1N1)pdm09	30.47	0	0	29.33	0
InfA_11	A(H1N1)pdm09	30.88	0	0	30.13	0
InfA_12	A(H1N1)pdm09	30.11	0	0	29.50	0
InfA_13	A(H1N1)pdm09	29.72	0	0	29.05	0
InfA_14	A(H1N1)pdm09	32.73	0	0	29.34	0
InfA_15	A(H1N1)pdm09	24.28	0	0	24.50	0
InfA_16	H3N2	28.46	0	0	28.30	0
InfA_17	H3N2	25.55	0	0	25.20	0
InfA_18	H3N2	22.93	0	0	22.36	0
InfA_19	H3N2	20.75	0	0	20.54	0
InfA_20	H3N2	29.43	0	0	29.08	0
InfA_21	H3N2	29.17	0	0	28.60	0
InfA_22	H3N2	30.52	0	0	30.22	0
InfA_23	H3N2	29.07	0	0	29.34	0
InfA_24	H3N2	26.91	0	0	27.13	0
InfA_25	H3N2	22.29	0	0	22.05	0
InfA_26	H3N2	25.44	0	0	25.60	0
InfA_27	H3N2	20.53	0	0	20.30	0
InfA_28	H3N2	25.97	0	0	25.17	0
InfA_29	H3N2	20.04	0	0	20.52	0
InfA_30	H3N2	23.52	0	0	23.32	0
Influenza B						
InfB_1	B/Vic	0	23.61	0	0	22.77
InfB_2	B/Vic	0	26.70	0	0	26.39
InfB_3	B/Vic	0	27.78	0	0	27.09
InfB_4	B/Vic	0	22.35	0	0	21.64
InfB_5	B/Vic	0	19.11	0	0	18.14
InfB_6	B/Vic	0	25.29	0	0	24.85
InfB_7	B/Vic	0	20.40	0	0	19.55
InfB_8	B/Vic	0	24.59	0	0	24.22
InfB_9	B/Vic	0	22.32	0	0	20.39
InfB_10	B/Vic	0	19.42	0	0	17.68
InfB_11	B/Vic	0	23.73	0	0	23.06
InfB_12	B/Vic	0	26.89	0	0	25.85
InfB_13	B/Vic	0	22.58	0	0	22.03
InfB_14	B/Vic	0	23.00	0	0	22.25
InfB_15	B/Vic	0	26.23	0	0	25.11
InfB_16	B/Vic	0	25.67	0	0	25.88
InfB_17	B/Vic	0	21.79	0	0	20.75
InfB_18	B/Vic	0	24.24	0	0	22.97
InfB_19	B/Yam	0	23.56	0	0	22.76
InfB_20	B/Yam	0	26.34	0	0	25.87
InfB_21	B/Yam	0	23.05	0	0	22.38
InfB_22	B/Yam	0	27.67	0	0	27.52
InfB_23	B/Yam	0	25.39	0	0	25.20
InfB_24	B/Yam	0	27.83	0	0	27.50
InfB_25	B/Yam	0	34.36	0	0	35.48
InfB_26	B/Yam	0	24.43	0	0	24.01
InfB_27	B/Yam	0	30.65	0	0	30.63
InfB_28	B/Yam	0	20.40	0	0	19.79
InfB_29	B/Yam	0	21.22	0	0	20.67
InfB_30	B/Yam	0	22.48	0	0	21.74

*Multiplex real-time reverse transcription PCR for influenza A virus, influenza B virus, and SARS-CoV-2. Singleplex assays comprise the CDC Flu rRT-PCR Dx Panel (*9,15*). B/Vic, B(Victoria); B/Yam, B(Yamaga); SARS-CoV-2, severe acute respiratory syndrome coronavirus 2.

Appendix Table 9. Comparison of the Influenza SARS-CoV-2 Multiplex Assay and singleplex assays using clinical SARS-CoV-2 specimens*

		Cycle threshold value					
		Multiplex				Singleplex	
Sample ID	Specimen type	Influenza A	Influenza B	SARS-CoV-2	RNase P (human)	N1	RNase P (human)
1	Throat/NP	0	0	28.68	23.22	29.16	22.62
2	NP	0	0	24.36	22.80	24.72	22.91
3	NP	0	0	15.27	24.02	15.16	23.35
4	NP	0	0	28.76	23.55	28.63	22.46
5	NP	0	0	24.67	24.33	24.56	23.74
6	Throat/NP	0	0	22.05	26.18	22.20	25.50
7	NP/Oral	0	0	23.07	24.36	23.06	24.08
8	NP	0	0	14.88	22.27	15.24	22.02
9	Throat/NP	0	0	20.52	23.26	20.40	22.52
10	NP	0	0	15.41	34.94	15.84	29.61
11	NP	0	0	25.23	30.57	25.57	29.80
12	NP	0	0	27.86	24.14	28.54	23.37
13	NP	0	0	18.02	23.26	17.73	22.90
14	NP	0	0	18.00	25.25	18.08	25.00
15	NP	0	0	21.24	24.25	21.29	23.73
16	NP	0	0	15.06	22.68	15.39	22.54
17	Sputum	0	0	30.61	23.55	31.18	22.82
18	Sputum	0	0	27.37	21.78	26.69	21.12
19	Sputum	0	0	14.78	23.69	14.34	21.47
20	NP	0	0	30.60	25.06	29.20	24.56
21	OP	0	0	30.50	30.47	29.49	30.16
22	NP	0	0	23.03	22.28	21.92	21.99
23	NP	0	0	31.43	23.36	31.77	22.97
24	NP	0	0	18.02	22.18	17.89	22.01
25	NP	0	0	18.12	22.35	18.17	22.29
26	Oral swab	0	0	25.11	27.53	25.61	27.46
27	Oral swab	0	0	25.45	30.83	25.82	29.97
28	Oral swab	0	0	24.20	25.61	25.05	25.16
29	Oral swab	0	0	22.51	26.09	23.38	25.44
30	Oral swab	0	0	19.31	26.12	19.68	26.26
31	Oral swab	0	0	24.88	26.75	25.24	26.88
32	NP	0	0	25.65	22.09	25.31	21.87
33	Oral swab	0	0	24.57	29.43	25.10	28.86

*Multiplex real-time reverse transcription PCR for influenza A virus, influenza B virus, and SARS-CoV-2. Singleplex assays comprise the CDC 2019-nCoV Real-Time RT-PCR Diagnostic Panel (7). NP, nasopharyngeal swab; OP, oropharyngeal swab; SARS-CoV-2, severe acute respiratory syndrome coronavirus 2.

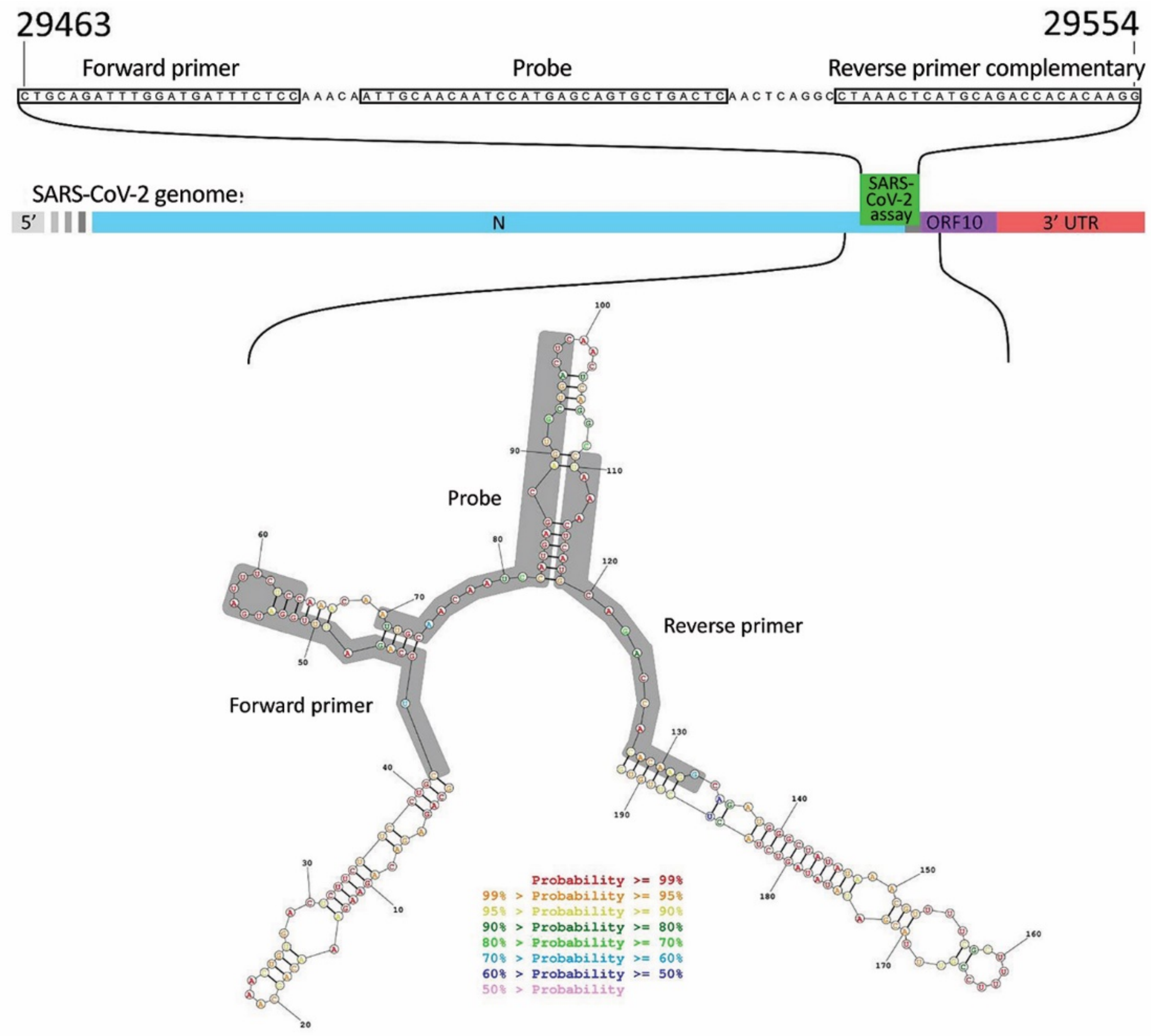

Appendix Figure. Relative genomic position of SARS-CoV-2 target used by the Influenza SARS-CoV-2 Multiplex Assay. N, nucleocapsid protein gene; ORF10, open reading frame 10; SARS-CoV-2, severe acute respiratory syndrome coronavirus 2; UTR, untranslated region.
